# Sol-Gel Composites-Based Flexible and Transparent Amorphous Indium Gallium Zinc Oxide Thin-Film Synaptic Transistors for Wearable Intelligent Electronics

**DOI:** 10.3390/molecules26237233

**Published:** 2021-11-29

**Authors:** Jin-Gi Min, Won-Ju Cho

**Affiliations:** Department of Electronic Materials Engineering, Kwangwoon University, Gwangun-ro 20, Nowon-gu, Seoul 01897, Korea; wlsrl1659@naver.com

**Keywords:** chitosan, microwave annealing, flexible substrate, solution-processed *a*-IGZO channel, synaptic transistors

## Abstract

In this study, we propose the fabrication of sol-gel composite-based flexible and transparent synaptic transistors on polyimide (PI) substrates. Because a low thermal budget process is essential for the implementation of high-performance synaptic transistors on flexible PI substrates, microwave annealing (MWA) as a heat treatment process suitable for thermally vulnerable substrates was employed and compared to conventional thermal annealing (CTA). In addition, a solution-processed wide-bandgap amorphous In-Ga-Zn (2:1:1) oxide (*a*-IGZO) channel, an organic polymer chitosan electrolyte-based electric double layer (EDL), and a high-*k* Ta_2_O_5_ thin-film dielectric layer were applied to achieve high flexibility and transparency. The essential synaptic plasticity of the flexible and transparent synaptic transistors fabricated with the MWA process was demonstrated by single spike, paired-pulse facilitation, multi-spike facilitation excitatory post-synaptic current (EPSC), and three-cycle evaluation of potentiation and depression behaviors. Furthermore, we verified the mechanical robustness of the fabricated device through repeated bending tests and demonstrated that the electrical properties were stably maintained. As a result, the proposed sol-gel composite-based synaptic transistors are expected to serve as transparent and flexible intelligent electronic devices capable of stable neural operation.

## 1. Introduction

The von Neumann architecture poses significant challenges in the post-Moore era, processing large amounts of data and diverse information while providing ever-increasing computing power with low energy consumption [1,2,3]. To overcome these problems, extensive studies on neuromorphic computing systems that mimic the behavior of the human brain have been conducted [4,5,6]. Among the various computing systems that mimic human brain behavior, synaptic transistors are considered one of the most fundamental building blocks of neuromorphic chips [7]. Neuromorphic chips with integrated memory have the processing capacity to conduct large-scale parallel and distributed operations using low power energy [8,9]. To realize these properties, an electric double layer (EDL) is frequently used as the gate dielectric layer in synaptic transistors. In particular, chitosan, a biodegradable, renewable, and biologically evolved material, is attractive among many EDL candidates [10]. In addition, high gate capacitance (>1.0 F/cm^2^) can be easily achieved from high-density mobile protons owing to the EDL effect of proton-conductive chitosan electrolytes that allow synaptic behavior [11,12]. However, despite these advantages, chitosan electrolytes have limited processing capabilities owing to their low chemical/mechanical resistance and ambient instability, which are expected drawbacks of organic materials. To solve these problems, recent studies have focused on the hybridization of organic and inorganic materials. In particular, Ta_2_O_5_ is a biocompatible high-k material, and when used as a barrier to the chitosan EDL layer, it stabilizes the chemical resistance and improves the mechanical strength, allowing compatibility with the CMOS process of the chitosan layer [13,14,15]. Meanwhile, the rapid advancement of display technology has accelerated the popularization of transparent and flexible electronic devices, which are expected to fulfill future technological requirements that are difficult to achieve with silicon-based electronic devices [16]. Therefore, both transparent and flexible channels and substrates for manufacturing electronic devices and configuring electronic circuits are essential. In general, prospective channel materials for transparent and flexible thin-film transistors (TFTs) include amorphous silicon (a-Si:H), metal–oxide semiconductors, and organic semiconductors [17,18]. In particular, metal-oxide semiconductors have 10 times higher mobility than a-Si:H and are compatible with the current TFT production workflows. Moreover, they are preferred because of their relatively better stability and electrical performance than their organic counterparts, as well as their low cost and a solution/printing process for mass production. On the other hand, considerable research has been conducted on flexible substrate materials such as polymer plastics, ultra-thin glass (UTG), and metal foil [16,19,20,21,22,23]. Polyimide (PI) is a widely used material owing to its low cost, excellent mechanical/chemical properties, and compatibility at high process temperatures [24,25,26,27,28]. Although PI withstands higher processing temperatures than other plastic substrates, the inherent thermal limitations of polymers cause damage at high temperatures, limiting their applications in flexible electronics or displays [27,28,29,30]. Therefore, there is a need for a heat treatment technique to improve the electrical properties of the device while preventing thermal damage to the flexible substrate. Microwave annealing (MWA) is an attractive technology in which materials interact with electromagnetic waves, volumetrically absorb electromagnetic energy, and convert it into heat. Because the sample is directly heated by energy transfer rather than heat transfer, MWA is a cost-effective heat treatment method with no energy wastage. In addition, the heat ramp-up and ramp-down time of MWA is rapid, which allows the main heat treatment processes to be performed in minutes, significantly reducing thermal budgets [31,32]. 

In this study, we fabricated sol-gel composite-based flexible and transparent amorphous In-Ga-Zn-oxide (*a*-IGZO) thin-film synaptic transistors on PI substrates. A solution-processed *a*-IGZO channel, an organic polymer chitosan electrolyte-based EDL, and a high-*k* Ta_2_O_5_ thin-film dielectric barrier layer was applied. To fabricate the synaptic transistors without thermal damage to the transparent and flexible PI substrate, MWA with a low thermal budget was applied. The essential synaptic plasticity of flexible and transparent synaptic transistors was evaluated by single spike, paired-pulse facilitation, multi-spike facilitation EPSC, and three-cycle evaluation of potentiation and depression behaviors. In addition, the mechanical robustness and electrical stability of the fabricated synaptic device were verified through repeated bending tests. 

## 2. Materials and Methods

Prior to synaptic transistor fabrication, the PI substrate was spin-coated on rigid glass with a thickness of 6 μm. Then using a plasma-enhanced chemical vapor deposition method, a 100/100 nm thick SiN_x_/SiO_2_ protective layer was formed on the PI substrate to avoid chemical damage. The PI substrate was annealed at various microwave powers and furnace temperatures to analyze the effect of MWA on the PI substrate and compare the thermal damage with conventional thermal annealing (CTA). The optical transmittance of the heat-treated PI substrate was measured using an Agilent 8453 UV-visible spectrophotometer (Hewlett-Packard Co., Palo Alto, CA, USA) to identify the thermal damage according to the heat treatment conditions. 

To fabricate the synaptic transistors, IGZO precursor solutions were prepared using a sol-gel process. At a molar ratio of 2:1:1, indium nitrate hydrate (In(NO_3_)_3_∙*x*H_2_O), gallium nitrate hydrate (Ga(NO_3_)_3_∙*x*H_2_O), and zinc acetate dehydrate (Zn(CH_3_COO)_2_∙*x*H_2_O) were dissolved in 2-methoxyethanol (C_3_H_8_O_2_) solvent, and mono-ethanolamine [O_2_H_7_NO] was added as a stabilizer. In a closed vessel, the mixed solution was agitated with a magnetic rod at 50 °C for 2 h, aged at room temperature for 24 h, and then filtered using a 0.2 μm syringe filter. For the TFT channel layer, the prepared IGZO (In_2_O_3_:Ga_2_O_3_:ZnO = 2:1:1 mol.%) precursor solution was spin-coated on the PI substrates at 6000 rpm for 30 s. Subsequently, the spin coated IGZO thin films were baked at 180 °C for 10 min to remove the solvent and impurities, and active channel regions (post-synapse) were formed using photolithography patterning and wet etching using a 30:1 buffered oxide etchant (BOE). Post-deposition annealing (PDA) to improve the electrical properties of the *a*-IGZO thin film was performed, where MWA with a frequency of 2.45 GHz at 1800 W was carried out in ambient air for 2 min, which is the optimal condition for excellent TFT properties. A 150-nm-thick Ti film was deposited using an electron beam (E-beam) evaporator, and the source and drain (S/D) electrodes were formed by a lift-off method. The chitosan electrolytic film, the main material of the proposed synaptic transistor, was spin-coated at 6000 rpm for 30 s, dried in air for 24 h, and then oven baked at 130 °C for 10 min, yielding a layer with a uniform thickness of 130 ± 5 nm. For the chemical/mechanical barrier layer, an 80-nm-thick high-*k* Ta_2_O_5_ layer was deposited by radio frequency (RF) magnetron sputtering at a working pressure of 4.0 mTorr, an RF power of 75 W, and an Ar flow rate of 20 sccm. The gate electrode (pre-synapse) was formed by depositing an Al film with a thickness of 150 nm using an E-beam evaporator, followed by a lift-off process. Finally, a reactive ion etching (RIE) procedure was performed to open the S/D contact holes. 

**Figure 1:**
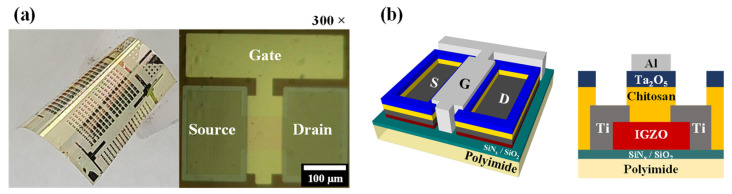
(**a**) Photograph and optical microscope image (300× magnification) of the sol-gel composites-based synaptic transistors. (**b**) Schematic diagram of sol-gel composites-based synaptic transistors.

Figure 1a,b show photographs and optical microscope images (300× magnification), and schematic diagrams of synaptic transistors based on flexible sol-gel composites, respectively. The neuromorphic characteristics, such as transfer curves, hysteresis window, threshold voltage (V_th_), single spike, paired-pulse facilitation (PPF), and multi-spike facilitation excitatory post-synaptic current (EPSC), were measured using an Agilent 4156 B precision semiconductor parameter analyzer. The pre-synaptic spikes and electrical pulses were applied using an Agilent 8110A pulse generator. In addition, potentiation and depression behaviors were assessed to monitor changes in synaptic weights. The synaptic device measurements were performed in a protective metallic dark shield box to avoid external influences, such as light and electrical noise. 

**Figure 2:**
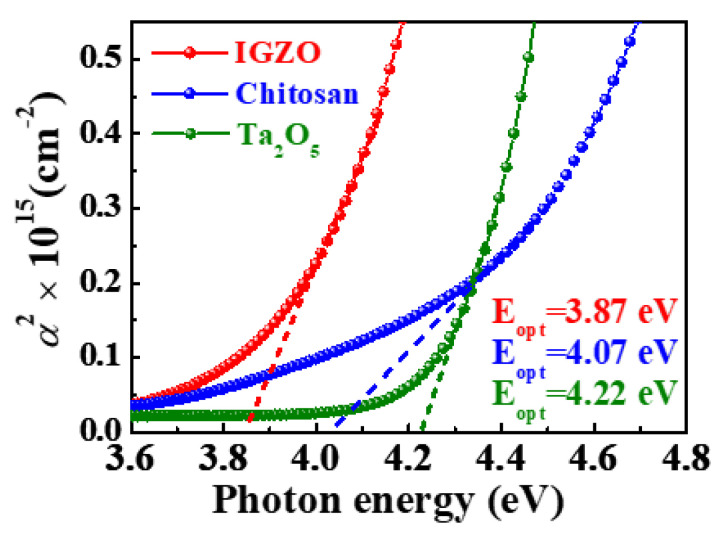
Optical band gap of the *a*-IGZO, chitosan, and Ta_2_O_5_ layers constituting sol-gel composites-based synaptic transistors.

Figure 2 shows the optical band gaps of *a*-IGZO, chitosan, and Ta_2_O_5_ layers constituting the sol-gel composite-based synaptic transistors extracted from Tauc plots using the following equation:

(1) $$a\left(E\right)=-\frac{1}{d}\ln\left(T_{normalized}\left(E\right)\right)$$

The optical band gaps for the *a*-IGZO, chitosan, and Ta_2_O_5_ layers are 3.87, 4.07, and 4.22 eV, respectively. Accordingly, the transparency of the sol-gel composite-based synaptic transistors has been demonstrated because each layer has a higher optical band gap than the band gap of visible light. 

## 3. Result and Discussion

**Figure 3:**
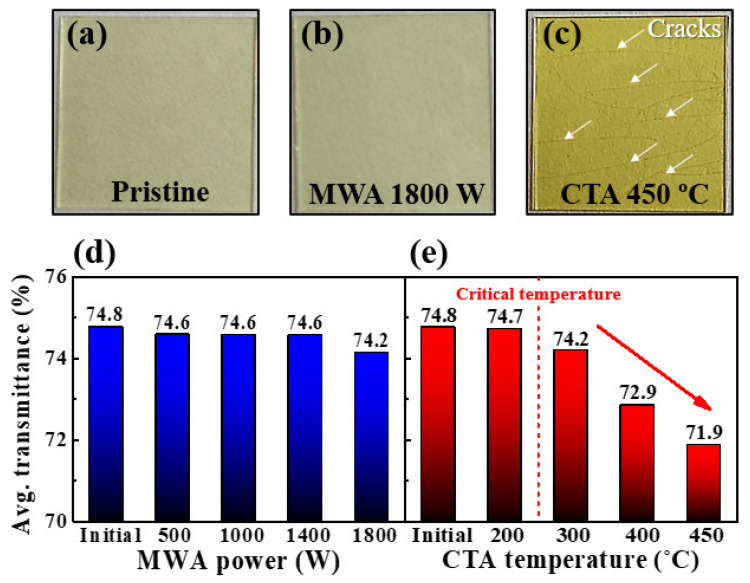
Photographs of thermal damage to the PI substrates during the annealing processes: (**a**) pristine, (**b**) MWA 1800 W, and (**c**) CTA 450 °C. Average transmittance in visible light (380–800 nm) as a function of (**d**) MWA power and (**e**) CTA temperature.

Figure 3 shows a photograph and the average transmittance of the pristine, MWA-, and CTA-treated PI substrates. It can be seen that the pristine PI substrates (Figure 3a) and those that were MWA-treated at 1800 W for 2 min (Figure 3b) are nearly identical. On the other hand, the PI substrate treated with CTA at 450 °C showed discoloration and severe cracks due to thermal damage (Figure 3c). Figure 3d,e show the average transmittance of the MWA- and CTA-treated PI substrates in the visible (380–800 nm) region, respectively. It is worth noting that the optical transmittance remained almost constant for MWA despite increasing the microwave power but decreased significantly with temperature from 300 °C for CTA [33]. Typically, solution-processed a-IGZO channels require heat treatment at temperatures significantly higher than 300 °C, but CTA prevents the PDA process because of the thermal damage to the PI substrate (Figure 3). Based on these results, we noted that MWA is an excellent heat treatment method for thermally vulnerable PI substrates and applied it with a low thermal budget as the PDA process for the solution-deposited *a*-IGZO films. 

**Figure 4:**
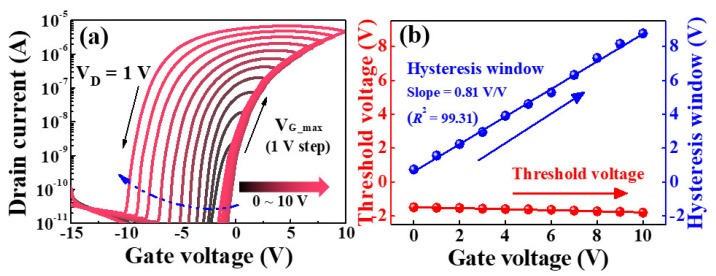
(**a**) Double−sweep transfer curves, and (**b**) hysteresis window and threshold voltage characteristics as a function of maximum gate sweep voltage.

Figure 4a shows the transfer characteristics (I_D_-V_G_) of the synaptic transistor measured by double-sweeping the gate voltage (V_G_). For the measurement of the double-sweep transfer characteristics, at a constant drain voltage (V_D_) of 1 V, the maximum gate bias (V_G_max_) increased positively (forward) by 1 V from 0 to 10 V, and then swept back negatively (reverse). It can be seen that the counterclockwise hysteresis appears owing to the V_G_ double-sweep, and the hysteresis window increases according to the V_G_max_ sweep range. The counterclockwise hysteresis window occurs owing to the slow polarization response of the mobile ions in the chitosan electrolytes [34]. Figure 4b shows the hysteresis window and threshold voltage (V_th_) as functions of V_G_max_. As V_G_max_ increased from 0 to 10 V, the hysteresis window linearly expanded from 0.74 to 8.17 V with a slope of 0.81 V/V and high linearity (R^2^ = 99.31), but V_th_ remained almost constant. Charge carriers are generated at the IGZO channel/hybrid-type EDL interface by the polarization of dipoles in the Ta_2_O_5_ high-*k* dielectric and migration of mobile ions in the chitosan electrolyte. Dipole alignment and migration of mobile ions can also occur because of changes in the gate electric field. The larger the value of V_G_max_, the stronger the electric field, dipole alignment, and ion accumulation, leading to a constant increase in the hysteresis window. These results make it difficult to swiftly restore the original state. Therefore, the hysteresis of the transfer curves increased in the double-sweep mode owing to the decrease in V_th_ and the increase in the drain current (I_D_). This phenomenon is initialized by applying a large negative V_G_, whereby the I_D_ of the sol-gel composite-based synaptic transistor returns to its starting value. The initialization features and linearity of the V_G_max_ versus hysteresis window of the suggested devices imply that they can emulate a biological synapse [35,36,37]. 

**Figure 5:**
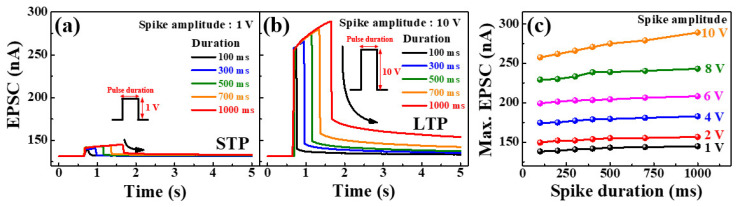
Single spike excitatory post-synaptic current (EPSC) curves of the sol-gel composites-based synaptic transistors with amplitudes of (**a**) 1 V and (**b**) 10 V for various pulse durations. (**c**) Maximum EPSC with various spike durations and amplitudes.

The gate voltage and channel conductance of the synaptic transistors are represented as the presynaptic stimulation and synaptic weights, respectively. The basic neuromorphic property of the synaptic transistors is the EPSC, induced by a single synaptic spike. Furthermore, repetition of a single synaptic spike affects post-synaptic short-term plasticity (STP), long-term plasticity (LTP), and long-term weight generation [38]. Figure 5 shows the single-spike EPSC curves of the sol-gel composite-based synaptic transistors with pulse amplitudes of (a) 1 V and (b) 10 V for pulse durations of 100 to 1000 ms. Figure 5c shows the maximum EPSC with various spike durations and amplitudes. If the spike amplitude was low and the duration was short, the EPSC value was low; however, the EPSC was sustained by the gradual polarization of the mobile protons inside the chitosan EDL. On the other hand, the higher the single spike amplitude and the longer the duration, the higher the EPSC value. In addition, the solution-processed *a*-IGZO channel layer was partially penetrated by the mobile protons, thereby increasing the magnitude of the residual EPSC in proportion to the amplitude and duration of the single spike. This indicates that electrochemical doping of solution-processed *a*-IGZO channels enhances channel conductivity and prolongs the resting time, indicating that synaptic weights can be controlled from STP to LTP [39]. Thus, the stronger and longer the spike stimulus, the greater the weight capacity to simulate human brain operation. Consequently, modulation of the EPSC by two or more repeated spikes is critical for biological systems to decode temporal information [40].

**Figure 6:**
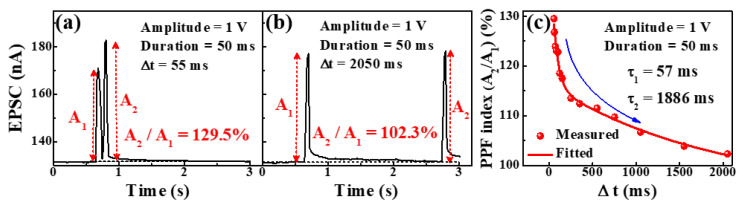
Paired-pulse facilitated excitatory post-synaptic current (EPSC) for (**a**) 55 ms and (**b**) 2050 ms intervals under the pulse amplitude of 1 V and duration of 50 ms. (**c**) PPF index with ∆t ranging from 55 ms to 2050 ms.

PPF is important for controlling synaptic plasticity in biological neural systems. The second synaptic spike produces higher post-synaptic potentials or currents as a function of the spike time interval (t) than the first synaptic spike [41,42]. Figure 6a,b show EPSCs triggered by paired consecutive presynaptic spikes (1 V, 50 ms) with interval times of 55 ms and 2050 ms, respectively. Incompletely relaxed protonic mobile ions make the second EPSC peak (A_2_) larger than the first EPSC peak (A_1_), and the mobile ions continue to accumulate near the interface [43]. Figure 6c shows the PPF index (A_2_/A_1_), calculated as the ratio between the two EPSC peaks. At the 55 ms interval, the PPF index was ~130%, but when the interval time was sufficiently long (t > 2050 ms), the PPF index decreased to ~102%. The measured PPF index data were fitted with the following double exponential decay relationship [44,45]:

(2) $$PPF\;index=A+C_{1}exp\left(-\frac{\Delta t}{\tau_{1}}\right)+C_{2}\;exp\left(-\frac{\Delta t}{\tau_{2}}\right)$$

where *A* is a constant, *C_1_* and *C_2_* are the initial facilitation magnitudes, and τ_1_ and τ_2_ are the characteristic relaxation periods. The PPF exponential decay process is well-fitted by the double-exponential decay relation, as shown by the solid lines. In our suggested synaptic transistors, τ_1_ and τ_2_ were 57 and 1886 ms, respectively. 

**Figure 7:**
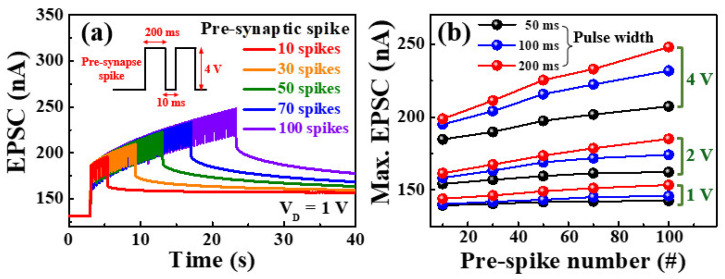
(**a**) Excitatory post-synaptic current (EPSC) facilitated by pre-synaptic stimulation of multiple pre-synaptic stimulation spikes (0–100 cycles). (**b**) Maximum EPSC according to pre-spikes number.

For the EPSC measurement of multiple presynaptic stimulation spikes, a pre-synaptic spike (4 V, 200 ms) was applied to the gate electrode, and a read voltage (V_D_ = 1V) was applied to the S/D electrode; the responses are shown in Figure 7a. The EPSC increased as the number of spikes applied to the gate increased, and then gradually decreased over time after the last spike. Figure 7b shows the change in the maximum EPSC according to the number of pre-spikes and the magnitude of the gate voltage. The maximum EPSC gradually increased as the number of pre-spikes (gate pulses) and gate voltage increased. In addition, the higher the maximum EPSC, the larger is the residual EPSC value. This suggests that by increasing the number of pulses and gate voltage, more mobile proton ions accumulated at the interface between the chitosan electrolyte and the solution-processed *a*-IGZO layer. This suggests that it takes longer to reach the equilibrium state, indicating LTP characteristics [10,41,46].

**Figure 8:**
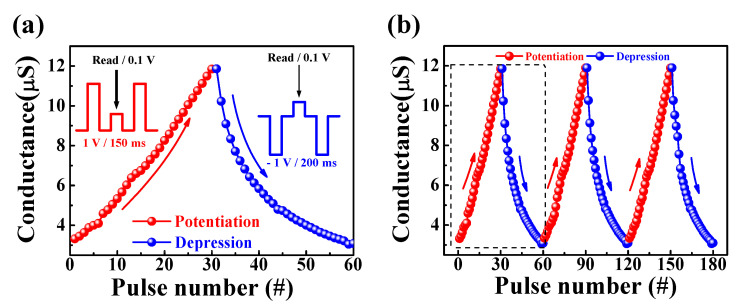
(**a**) Potentiation and depression behaviors by applying pre−synapse pules with conditions of 1 V for 150 ms and −1 V for 200 ms. (**b**) Three−cycle endurance of potentiation and depression behaviors.

Figure 8a shows the conductance modulation of 30 potentiation (red circles) and 30 depression (blue circles) pre-synaptic pulses. Pulse patterns for potentiation, depression, and read voltage are shown in Figure 8a. One cycle included 30 potentiation pulses and 30 depression pulses with pulse conditions of 1 V for 150 ms and −1 V for 200 ms. The dynamic range of conductance is from 3.32 μS to 11.85 μS for the proposed synaptic transistors. The endurance of conductance when these potentiation and depression behaviors were repeated three times is shown in Figure 8b, where the conductance modulation remained nearly constant for the three cycles. We also evaluated synaptic weight values in response to stimulation with the potentiation/depression pulses. These results demonstrate that learning processes can be performed in artificial neural networks, indicating their applicability to future artificial synaptic devices [47].

**Figure 9:**
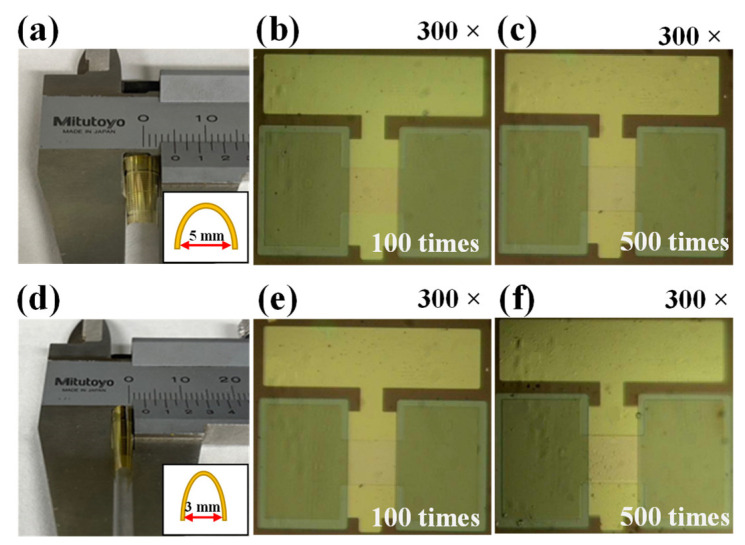
Polyimide substrates on which sol-gel composite-based synaptic transistor devices were formed using vernier calipers bent to (**a**) 5 mm and (**d**) 3 mm in diameter. Optical microscopy images of the sol-gel composite-based synaptic transistor devices after (**b**) 100 times and (**c**) 500 bending tests with a diameter of 5 mm, and after (**e**) 100 times and (**f**) 500 times bending tests with a diameter of 3 mm.

Figure 9 shows the results of the bending endurance test of the PI substrate treated with MWA at 1800 W for 2 min to verify its mechanical strength. Figure 9a–c are the bent images of the PI substrate and optical microscopy images of the sol-gel composite-based synaptic transistors after 100 bending tests, while Figure 9d–f show the results after 500 bending tests. Using the most basic and widely used bend radius tests, the flexibility of the PI films was evaluated with a bending diameter of 5 mm and 3 mm. Both the MWA-treated PI substrates and sol-gel composite-based synaptic transistors exhibit excellent bending durability without mechanical damage at 5 mm and 3 mm bending diameters. This is due to the selective heating properties of MWA, where only the sol-gel composite material-based synaptic transistor is subjected to the PDA processing, while the PI substrate remains unheated. Therefore, based on this unique heating behavior, we conclude that MWA is a suitable heat treatment approach for fabricating transparent and flexible electronics on PI substrates.

**Figure 10:**
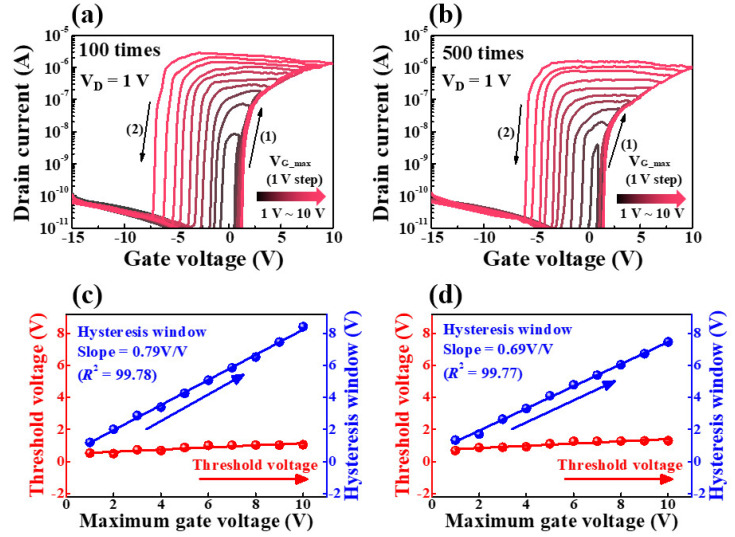
Double−sweep transfer curves as a function of V_G_max_ after bending (**a**) 100 times and (**b**) 500 times to 3 mm. Hysteresis window and threshold voltage characteristics as a function of maximum gate sweep voltage after bending (**c**) 100 and (**d**) 500 times to 3 mm.

The effect of mechanical stress on the electrical properties of the sol-gel composite-based synaptic transistor was evaluated by measuring the transfer curve after bending the device 100 or 500 times at a bending radius of 3 mm. Figure 10a,b show the double-sweep transfer curves as a function of the maximum gate sweep voltage after 100 and 500 bends to 3 mm, respectively. The sol-gel composite-based synaptic transistor exhibited n-type transistor performance, even after the bending test. Figure 10c,d show the hysteresis window and V_th_ extracted from the transfer curves of the V_G_max_ after 100 and 500 bends to 3 mm, respectively. After 100 bending cycles, the hysteresis window linearly increased from 1.19 V to 8.43 V with 0.79 V/V slope and high linearity (R^2^ = 99.78), while after 500 bending cycles, the hysteresis window linearly increased from 1.33 V to 7.49 V with a 0.69 V/V slope and high linearity (R^2^ = 99.77). Therefore, it is apparent that V_th_ remains almost constant with respect to the increase in V_G_max_ even after 100 and 500 bending tests. Meanwhile, the hysteresis window became slightly smaller as the number of bending cycles increased. Therefore, MWA was found to be a thermally damage-free process on thermally vulnerable PI substrates. Furthermore, the sol-gel composite-based synaptic transistors on the PI substrates fabricated by applying MWA demonstrated excellent mechanical reliability and stable electrical properties even after the bending tests. 

## 4. Conclusions

We fabricated high-performance sol-gel composite-based *a*-IGZO synaptic transistors on transparent and flexible PI substrates by using a low thermal budget MWA method. We were able to establish a low thermal budget condition, which is essential for realizing high-performance synaptic transistors, by comparing the process suitability of MWA with that of CTA. In particular, the thermally susceptible PI film cracked under CTA at 450 °C; however, the PI substrates that were heat-treated with MWA 1800 W were damage-free. In addition, a solution-processed *a*-IGZO channel layer, organic polymer chitosan electrolyte, and high-*k* Ta_2_O_5_ thin-film dielectric layer were used to impart high flexibility and transparency. Furthermore, we evaluated vital synaptic characteristics such as single spike, paired-pulse facilitation, multi-spike facilitation EPSC, and conductance evaluation of potentiation and depression behaviors. To identify the mechanical robustness, a repetitive bending test was performed, during which the electrical properties were also stably maintained. As a result, the proposed sol-gel composite-based *a*-IGZO synaptic transistors are prospective artificial electronics for the future owing to their stable synaptic operations.

## Data Availability

Not applicable.
